# Quality of Life in Painful Peripheral Neuropathies: A Systematic Review

**DOI:** 10.1155/2019/2091960

**Published:** 2019-05-23

**Authors:** Ayesha Girach, Thomas Henry Julian, Giustino Varrassi, Antonella Paladini, Athina Vadalouka, Panagiotis Zis

**Affiliations:** ^1^The Medical School, University of Sheffield, Sheffield, UK; ^2^Paolo Procacci Foundation, Via Tacito 7, Roma, Italy; ^3^Department of MESVA, University of L'Aquila, L'Aquila, Italy; ^4^Athens Medical Centre, Athens, Greece; ^5^Medical School, University of Cyprus, Nicosia, Cyprus; ^6^Academic Department of Neurosciences, Sheffield Teaching Hospitals NHS Trust, Sheffield, UK

## Abstract

**Objective:**

Neuropathic pain is a common presenting complaint of patients with peripheral neuropathy (PN) and is considered one of the most disabling neuropathic symptoms, with detrimental effects on patients' quality of life (QoL). The aim of this review was to overview the current literature that focuses on QoL in painful PN of various aetiologies. We sought to clarify the direct effect of pain and its treatment on patients' QoL.

**Methodology:**

A systematic computer-based literature search was conducted using the PubMed database to search for papers on QoL in painful PN. Information was extracted regarding prevalence, demographics, and response to treatment where relevant.

**Results:**

We identified 66 articles eligible for inclusion. The vast majority of studies (*n*=47) focused on patients with diabetic PN. Other aetiologies of painful PN where QoL has been studied to date include gluten, immune-mediated, HIV, chemotherapy-induced, and chronic idiopathic axonal polyneuropathy. Pharmacological treatment is the mainstay in managing pain and has a direct positive and independent effect on the overall QoL. Other nonpharmacological approaches can also be of benefit, either alone or as adjuvant treatments, and are discussed.

**Conclusion:**

The findings demonstrate that QoL is impaired in painful PN and should not be neglected in clinical practice. Patients' pain management and subsequent impact on QoL should routinely be assessed and monitored.

## 1. Introduction

The term “peripheral neuropathy” (PN) refers to disorder of the peripheral nervous system. Robust epidemiological data on polyneuropathies of any cause are lacking. Very few studies have accurately assessed the prevalence of polyneuropathy. When confirmed with nerve conduction studies, the prevalence of polyneuropathy is estimated to be about 5% in people aged 55 years or more [1]. Thus, polyneuropathy is considered to be a common neurological disease.

Classification of PN depends upon a mixture of phenomenological, neurophysiological, pathological, and aetiological parameters [2]. The commonest form of PN is the chronic axonal length-dependent sensorimotor polyneuropathy. Neuropathic symptoms can be divided into sensory and motor. Sensory symptoms include tingling; pins and needles; numbness; tightness; burning; pain; and sensory ataxia. Motor symptoms include muscle cramps, stiffness, weakness, and wasting [3].

Neuropathic pain is prevalent, presenting in approximately two-thirds of patients with PN [4–10] with minimal variability across its aetiological classifications, and is considered to be one of the most disabling neuropathic symptoms having a detrimental effect on patients' mental health [11] and leading to poor quality of life (QoL).

The aim of this study was to systematically review the current literature regarding QoL in patients with painful PN. We aimed to evaluate any variations in QoL between the different PN subtypes and more specifically clarify what is the direct effect of pain in patients' QoL. The effect of the various treatments on the overall QoL is also discussed. To our knowledge, this is the first systematic review on the topic.

## 2. Methodology

### 2.1. Search Strategy

A systematic computer-based literature search was conducted on 11 December, 2018, using the PubMed database. We evaluated all articles published between the dates of 1 January 1998 and 11 December 2018. For the search, we used three Medical Subject Headings (MeSH) terms that had to be present in the title or the abstract. Term A was “quality of life” or “qol.” Term B was “pain” or “painful.” Term C was “neuropathy” or “polyneuropathy” or “ganglionopathy” or “neuronopathy.” “Human species,” “English language,” and “full-text available” filters were applied in our search.

### 2.2. Inclusion and Exclusion Criteria

In order to be included in this review, articles were required to meet the following criteria: [1] original articles; [2] study human subjects; [3] written in English language; [4] refer to painful peripheral neuropathy; [5] refer to the QoL of subjects. The exclusion criteria for the articles were as follows: [1] book chapters, reviews, meta-analyses, letters to the editor, and editorials not providing new data and study protocols; [2] articles not referring to patients with painful peripheral neuropathies; [3] articles with a lack of individual results for the painful peripheral neuropathies, even if these subjects were included in the study; [4] articles which did not explore QoL as an outcome measure in sufficient depth.

### 2.3. Synthesis of Results

This study is reported in accordance with the “Preferred Reporting Items for Systematic Reviews and Meta-Analysis (PRISMA) guidelines” [12]. A database was developed using the Statistical Package for Social Science, version 24 for Mackintosh. Pooled frequencies and descriptive characteristics of demographic parameters were extracted.

### 2.4. Compliance with Ethical Guidelines

This article is based upon previously published studies. The article is in compliance with the journal's ethical guidelines.

## 3. Results

### 3.1. Selected Studies

**Figure 1:**
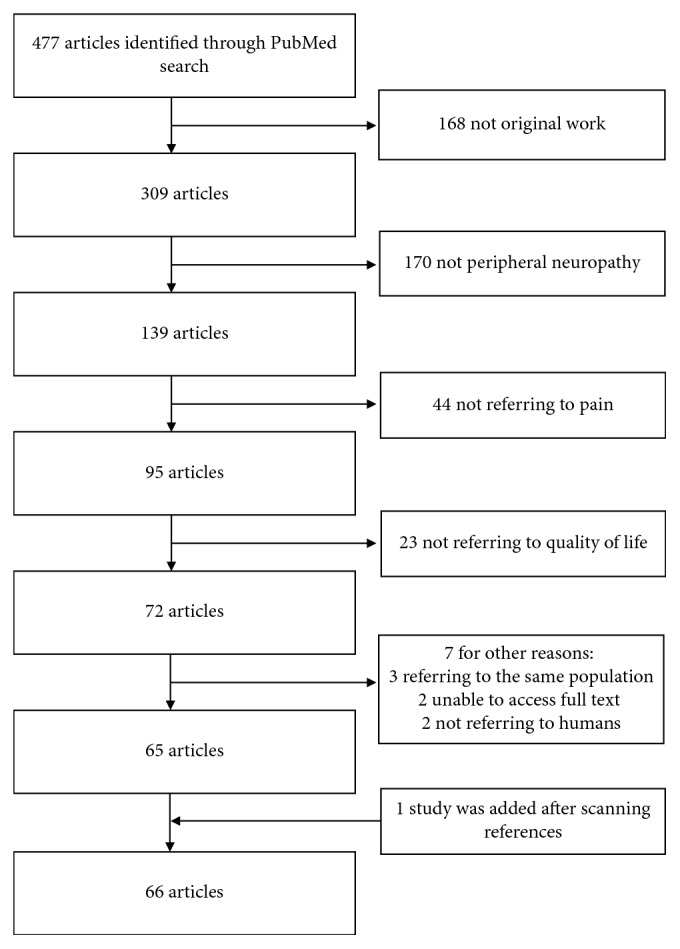
PRISMA chart.

The PubMed search identified 477 articles, and a total of 412 articles were excluded during the eligibility assessment. A further article was added after scanning the references of the included studies. The PRISMA chart displays the process of article selection (Figure 1). In total, 66 articles met the inclusion criteria. Case series constituted the commonest type of paper (45.5%), closely followed by randomised controlled trials (43.9%). The number of articles per decade since 1998 has rapidly increased, with the biggest increment between the years 2010 and 2018. The vast majority of articles were on diabetic PN (71.2%). A summary of article characteristics has been demonstrated in Table 1.

### 3.2. Assessment of QoL

In total, 19 different tools were used to assess QoL in PN patients. The most commonly utilised tools were the SF-36 (34.8%) and the EQ-5D (22.7%), both of which are generic health status measurements. A number of disease-specific questionnaires were employed, such as the NeuroQol (6.1%), a specifically validated neuropathy and foot ulcer instrument [13]. A tabulated breakdown of the named questionnaires used in articles included in this review is provided in Table 2.

### 3.3. Quality of Life across Painful Neuropathies of Different Aetiologies

A selection of studies examined aetiologically heterogeneous populations of patients with PN. The disability caused by PN has been shown to correlate with a decrease in QoL, and it has been demonstrated that painful PN is associated with a poorer QoL compared to painless PN, regardless of the cause [14–19]. The majority of the available literature, however, focuses on neuropathies of specific aetiologies, and therefore, we present the available knowledge per neuropathy type in the following.

#### 3.3.1. Diabetic Peripheral Neuropathy

QoL is one of the most important aspects of patients' lives affected by diabetes, both due to its effect on the long-term prognosis and on the economic burden of the disease.

*(1) Direct Effect of Pain*. Throughout the literature, numerous studies have evaluated the effect of pain on QoL and consistently found that those with painful diabetic peripheral neuropathy (DPN) have an impaired QoL, particularly in relation to their reduced physical activity [20–29]. In a large study conducted by Won et al., it was shown that patients with higher pain intensity experienced the worse QoL [28].

*(2) Socioeconomic Status*. In a study that was conducted by daCosta DiBonaventura et al., it was shown that patients with painful DPN not only have a significantly worse QoL compared to patients with painless DPN and healthy controls but also patients with painful DPN have more comorbidities and are from lower socioeconomic backgrounds [26]. Sufferers of painful DPN have often suffered from diabetes for a longer duration of time and lack compliance in management of their condition [26]. The financial implications of this, both due to direct and indirect costs, can lead further to a reduced QoL [26]. Direct costs include hospitalisations and healthcare provider visits, whilst indirect costs include timeoff work due to illness and additional care from family and friends to help manage their condition.

*(3) Sleep*. Sleeping disorders are common in painful DPN. A vicious cycle is established, as a lack of sleep can worsen the perception of pain, which as a result leads to an increased burden of disease [15, 22, 27, 29].

*(4) Mental Health*. Stress can exacerbate the pain perceived by patients with DPN, as it does for most patients with all types of chronic pain [27]. Pain will lead to higher stress levels, creating another vicious cycle, which leads to poorer QoL.

Depression and anxiety also have an impact on perceived QoL. Depressed patients may report lower QoL at baseline in clinical trials and subsequently negative treatment effects [11, 19, 29, 30]. Research has shown that painful DPN is associated with catastrophic and anxiety provoked thinking, which as a result leads to a perceived decline in physical activity and subsequent reduction in QoL [30, 31]. Geelen et al. illustrated that patients with DPN suffer from various fears including those of hypoglycaemia, negative evaluation, falling, and fatigue and some of these fears are associated with a reduced QoL and increased disability [32]. These observations are relevant to clinical practice, as they provide a theoretical framework on the psychosocial consequences of painful DPN, which can enable designing treatment strategies to address these specific fears. The same study group looked into pain catastrophizing, defined as a negative cognitive set brought about during actual or anticipated pain experience, and showed that it is associated with a decline in physical activity and an increased perception of disability and decreased QOL in patients with painful DPN [31].

*(5) Effect of Treatments on QoL*. A lack of curative treatment for DPN means that treatment approaches are aimed both at decelerating the disease's progression through better glycaemic control and at pain management [22, 33]. Evidence of earlier diagnosis of DPN and addressing foot care issues promptly can reduce the effect of the disease on QoL [20]. Schumacher et al. identified that other members of the multidisciplinary team can play an important role in the earlier identification of painful DPN and assist them in receiving the appropriate care [24].

*(6) Improving Glucose Control*. Rokicka et al. compared the effects of intravenous (the study group) versus subcutaneous (control group) delivery of insulin [34]. Both were comparable in terms of their reduction of pain in patients. A more intensified insulin regime resulted in an overall improved condition of general health although an improvement in QoL was only observed in control subjects.

*(7) Anticonvulsants*. Three randomised controlled trials looking at the effect of pregabalin compared to placebo found significant improvements in many aspects of QoL, including mental health and sleep, in patients who were refractory to previous treatments [35, 36].

Lacosamide has a long-term safety profile and sustained efficacy in PDN. Apart from treating pain, use of lacosamide leads to improvements in the “physical functioning” and “vitality” subdomains of the SF-36 [37, 38].

Gabapentin has proven to be an effective pain relief and has demonstrated additional benefits in improving patients sleep quality and mood [39].

*(8) Cannabinoids*. Conflicting evidence exists regarding the use of cannabinoids in painful conditions [17]. The first trial assessing the efficacy of a cannabis-based medicinal product (nabiximols) has shown it to be no more efficacious than placebo [40]. However, the results of this trial are contradictory to later findings, where the cannabinoid nabilone has been found to have similar benefits upon pain relief, sleep, and anxiety compared to gabapentin when used as a monotherapy as well as an adjuvant therapy [17]. Whilst the specific reason for this conflict is unclear, it should be noted that these are very different medications in that nabilone is a synthetic, oral capsule delivery cannabinoid, whilst nabiximols is a cannabis extract with oro-mucosal delivery and they do not have the same active compositions.

*(9) Antidepressants*. A Japanese-based study comparing 40 mg to 60 mg of duloxetine found that both doses are tolerable and effective and can be considered long-term treatment options for improving pain severity and QoL [41]. Furthermore, when in a study comparing duloxetine with pregabalin, despite both having a significant effect in reducing DPN pain, duloxetine was found to have better efficacy [42]. The cost-effective analysis determined that duloxetine was dominant to pregabalin as calculated using incremental cost-effectiveness ratio with QoL as the unit of outcome.

Amitriptyline and nortriptyline were found to have equal tolerability and efficacy in painful DPN both as monotherapy and as adjuvant therapies; however, no statistically significant changes were noted in overall QoL [43].

*(10) Opioids*. Tramadol is well established as being safe and effective in the treatment of painful DPN [44] and when given together with acetaminophen have shown improvements in pain intensity, QoL, mood, and function compared to placebo [45]. Similarly, controlled-release oxycodone is effective, safe, and has demonstrated significant improvements in QoL [46].

*(11) Topical Agents*. Capsaicin is the most studied topical agent for painful DPN and has proven beneficial in pain control as well as improving patient QoL [47, 48]. In a large, phase 3-randomised, 52-week study, capsaicin (8% patches) in addition to standard care of treatment was well tolerated and effective. The observed safety evaluations may have been biased by the open-label design of this study [47].

One, small, randomised controlled trial evaluated the application of topical *Citrullus colocyntis* fruit extract showing that it can decrease pain and may have some effect on the physical domain of QoL [48].

A double-blind randomised controlled trial found that topical nutmeg extracts reduce pain in painful DPN and improve overall QoL after 4 weeks of treatment; however, these effects were not superior to placebo [49].

*(12) Nutritional Supplementation*. Vitamin E supplementation has been found to have a significant role in controlling pain in DPN, leading to an overall improvement of QoL after prolonged treatment [50]. Alam et al. reported that the administration of a single high dose of Vitamin D showed significant improvements in the emotional distress subscale of the NeuroQol, in addition to improvements in pain-related symptom scores [13].

The effects of alpha-lipoic acid were found to have a clinically significant impact on controlling neuropathic pain and improving overall QoL in a cohort of patients taking this agent orally [51]. Similarly, LMF-MC-PP (L-methylfolate-methylcobalamin-pyridoxal-5-phosphate) is a nutritional treatment for DPN that was shown to be effective in reduction of pain intensity and improvement in QoL [52].

*(13) Surgical Interventions*. Patients with PN are prone to superimposed entrapment neuropathies, and in these cases, surgical decompression has been proven to significantly relieve pain [53], with patients' QoL also subsequently improving [54].

Spinal cord stimulation (SCS) is an invasive treatment for chronic pain, based on electrical stimulation of the dorsal columns [55]. It has proven to be an acceptable treatment modality in patients who do not respond to conventional medical treatment [55–58]. In an open-label study conducted by De Vos et al., as well as a reduction in intake of pain medication, SCS showed improvement on sleep that persisted for 6 months [58].

*(14) Other Nonpharmacological Treatments*. Despite the practice of mindfulness meditation showing to have a positive effect on physical and psychological outcome in varied patient populations, no significant improvements were noted in a pilot study looking at PDN, including QoL [33].

Low-frequency pulsed magnetic field magnetostimulation showed no advantage over sham exposure in reducing pain intensity, decreasing sleep disturbance, or improving QoL [59].

Findings from a randomised, double-blind, placebo controlled trial suggest that the administration of photon stimulation resulted in significant improvements in sensation, social functioning, and mental health; however, further studies are needed to investigate different doses and durations of treatment [60].

Although rarely addressed in the literature, wearing static, permanent magnetic insoles produces a significant reduction in pain, either as an adjunctive or monotherapy [61].

Aromatherapy massage has been found to be a safe, effective, low-risk treatment option with high compliance rates, with both pain and QoL scores significantly improving after 4 weeks of treatment [62]. However, this was a small (*n*=46), unblinded, noncontrolled study.

#### 3.3.2. Gluten Neuropathy

Peripheral neuropathy is a common extraintestinal manifestation of serologically confirmed gluten sensitivity (positive gliadin antibodies and/or tissue transglutaminase or endomysium antibodies) [7, 11, 63]. Up to 55% of patients with gluten neuropathy experience pain [64]. The resultant neuropathic pain is associated with poorer mental health status, and amelioration of overall pain and health status can be achieved with a gluten-free diet [7, 63, 64]. Zis et al. demonstrated that a strict gluten-free diet (as evidenced by the elimination of gluten sensitivity-related antibodies) results not only in better scores on the pain domain of the SF-36 but also in better scores on the overall health change domain (how patients perceive their overall health across time).

#### 3.3.3. Immune-Mediated Neuropathy

Anti-myelin associated glycoprotein antibody (anti-MAG) neuropathy is a type of immune-mediated neuropathy. Rajabally et al. showed that presence of pain has a significant impact on QoL. This study brings new insights on the practical management of patients with anti-MAG neuropathy, in indicating that neuropathic pain and pain related to cramps play a significant role in the impairment of function and the overall QoL in affected patients [53].

#### 3.3.4. Chemotherapy-Induced Peripheral Neuropathy

Many chemotherapy treatments induce peripheral neuropathy (CIPN), and it is a persistent problem beyond treatment [5, 65]. Results from a recent study have shown that painful CIPN independently affects the overall patient QoL although general health was rated as high.

Duloxetine has been tried as a treatment of painful CIPN in 2 studies [66, 67] and was found to have statistically and clinically significant improvements in pain and QoL when compared to placebo. In addition to exploring the analgesic effects of duloxetine, it has been shown that patients with better baseline emotional health, such as feeling less worried or anxious, are four times more likely to respond to duloxetine [66].

Opioids such as tapentadol have also been used in the treatment of CIPN. Galie et al. found QoL scores were significantly improved after treatment, thus proving a correlation between treating pain and subsequently QoL [68].

#### 3.3.5. HIV-Related Polyneuropathy

HIV-related polyneuropathy is prevalent in HIV patients; however, the exact cause is unknown. Hypothesized mechanisms include altered immunity, nutritional factors, infectious processes and as a result of the adverse effects of highly active antiretroviral therapy causing damage to the peripheral nervous system [69–71].

Regarding pharmacological therapies, although capsaicin is effective in treating pain associated with HIV-associated PN, it has not shown a significant improvement of the overall QoL [70, 72].

Nonpharmacological therapies include hypnosis, which has not only shown reduction in pain intensity but an improvement in QoL and a reduction in depression-related symptoms [71]. This benefit was found in patients for 7 weeks, irrespective of whether or not they were taking pain reduction medication.

Moreover, Knezevic et al. presented the first reports of spinal cord stimulation in two patients refractory to conservative treatments [69]. Both patients reported significant improvements in their ability to carry out daily activities, an overall increase in QoL, and a reduction in the use of opioid analgesics, demonstrating this to be a safe and viable treatment option. An important limitation to this would be ensuring the CD4 counts of potential recipients are within safe limits, as those who are severely immunosuppressed could increase their susceptibility to infection when undergoing spinal cord stimulation [69].

#### 3.3.6. Genetic Neuropathies

Only one study in our review evaluated the effect of painful Charcot–Marie–Tooth (CMT) disease (mainly CMT1 and CMT2) on QoL in a paediatric population and found pain negatively affects QoL [73].

#### 3.3.7. Haemodialysis-Related Neuropathy

PN is a common occurrence in haemodialysis patients and has been related to an impaired QoL. Reduced QoL is independently related to worse clinical outcomes and increased mortality in haemodialysis patients [16]. Atalay et al. found significant improvements in QoL in patients with PPN treated with gabapentin and pregabalin, through a reduction of pain intensity [16].

#### 3.3.8. Sarcoidosis-Related Neuropathy

Sarcoidosis is an inflammatory disease affecting many tissues, including dysfunction of small nerve fibres, the prevalence of which is grossly underestimated [74]. High-dose glucocorticoids have been the mainstay of treatment but however are associated with unacceptable side effects. In a randomised , double-blind, pilot study, Heij et al. demonstrated that ARA 290, a peptide designed to activate the innate repair receptor that initiates cytoprotection, anti-inflammation, and healing, is a safe and effective treatment option, including improvement in QoL [74].

#### 3.3.9. Chronic Idiopathic Axonal Polyneuropathy

Chronic Idiopathic axonal polyneuropathy (CIAP) is a term describing neuropathies with sensory and motor involvement, in a length-dependant distribution. It is slowly progressive, insidious, with no identifiable aetiology despite extensive diagnostic work-up [3, 75]. CIAP is correlated with a worse QoL in the energy/fatigue domain, the emotional well-being domain, and the general health perception domain [8]. In a small, open-label trial, lidocaine (5% patches) was found to significantly improve pain and QoL in CIAP [76].

## 4. Conclusions and Future Directions

This systematic review has identified the following key points:

- Pain has an additional negative impact on QoL in patients with PN, regardless of the aetiology of their neuropathy.
- The treatment of neuropathic pain is universally the same, and current guidelines for the treatment exist [77]. Treatment of pain can further ameliorate QoL.
- It has been highlighted that specific diets (based on the aetiology of the neuropathy) can play an additional role in improving QoL, for example, diet control in diabetes and a gluten-free diet in gluten neuropathy.
- This systematic review has highlighted that only one paper focuses on a paediatric population, where the cause of the neuropathy was CMT. In order to better understand the QoL in adult populations, further assessment of genetic neuropathies should be studied using a variety of population ages.
- It is likely that patients with PN suffer from comorbidities that are affecting the QoL independently to the burden caused by the painful peripheral neuropathy. Comparing the QoL of groups of patients with PN of different aetiologies using multivariate statistics can eliminate this risk of bias.
- The majority of the tools for evaluating QoL that were used in the papers included in this review were generic (i.e., SF-36). Designing and validating tools for evaluating QoL in patients with PN are important. Such tools should capture and weigh accordingly specific to PN domains that might be affected.

## Figures and Tables

**Table 1:** Characteristics of the papers included in the review.

Total number of papers included in this review	66

Type of paper (%)	
Case series	30 (45.5)
Case-controlled study	7 (10.6)
Randomised controlled trial	29 (43.9)
Mean number of patients with painful neuropathy, per paper (SD)	125.1 (122.5)
Male to female ratio	1 : 1

Aetiology of polyneuropathy, number of papers (%)	
Chronic idiopathic axonal	2 (3.0)
Chemotherapy-induced	4 (6.1)
Diabetic	47 (71.2)
Genetic	1 (1.5)
Gluten	1 (1.5)
HIV	3 (4.5)
Immune-mediated	1 (1.5)
Multiple aetiologies	6 (9.1)
Sarcoidosis	1 (1.5)

Number of publications per decade	
Until 2000	3 (4.5)
2000–2009	16 (24.2)
2010–2018	47 (71.2)

**Table 2:** Questionnaires used to assess quality of life.

Questionnaire	*N* (%)^*∗*^
Brief Pain Inventory	7 (10.6)
Child Health Questionnaire	1 (1.5)
Diabetic peripheral neuropathic pain impact measure	1 (1.5)
EQ-5D	15 (22.7)
Functional Assessment of Cancer Therapy/Gynecologic Oncology Group-Neurotoxicity (fact/GOG-ntx) Questionnaire	1 (1.5)
Medical Outcomes Study HIV Health Survey	1 (1.5)
Neuropathic Pain Impact on Quality of Life (NePIQoL) Questionnaire	1 (1.5)
NeuroQoL	4 (6.1)
Norfolk Quality of Life Questionnaire-DN	4 (6.1)
Nottingham Health Profile	1 (1.5)
PART-Q30	1 (1.5)
QLQ-C30	2 (3.0)
QLQ-CIPN20	1 (1.5)
Quality of life index	1 (1.5)
Questionnaire not specified/unvalidated	6 (9.1)
SF-12	2 (3.0)
SF-36	23 (34.8)
Sheehan disability score	2 (3.0)
World Health Organization Biomedical Research and Education Foundation quality of life score	1 (1.5)

^*∗*^A number of papers used more than one questionnaire.

## Additional Points

*Limitations*. (i) A more comprehensive search using other databases other than PubMed would have produced a greater number of articles eligible for final analysis. (ii) Many of the studies based a diagnosis of peripheral neuropathy on clinical interpretation or self-reported questionnaires. Consequently, selection bias may be present. A more robust and reliable diagnostic tool would be using neurophysiological studies throughout the studies.
